# Reduced Circulating MOTS-c Levels in Hashimoto’s Thyroiditis Reflect Integrated Autoimmune and Metabolic Dysregulation: A Cross-Sectional Study

**DOI:** 10.3390/jcm15114002

**Published:** 2026-05-22

**Authors:** Hanişe Ozkan Sonay, Eda Nur Duran, Murvet Algemi, Berrak Sahtiyanci, Irem Kirac Utku, Esra Çokiçli, Naile Fevziye Misirlioglu, Gonul Simsek, Hafize Uzun, Omur Tabak

**Affiliations:** 1 Department of Internal Medicine, Kanuni Sultan Suleyman Training and Research Hospital, Health Sciences University, 34303 Istanbul, Türkiye; haniseozkan@hotmail.com; 2 Department of Internal Medicine, Pervari State Hospital, 56700 Siirt, Türkiye; enurduran@gmail.com; 3 Department of Clinical Biochemistry, Kanuni Sultan Suleyman Training and Research Hospital, Health Sciences University, 34303 Istanbul, Türkiye; algemimurvet@gmail.com; 4 Department of Internal Medicine, Istanbul Physical Therapy and Rehabilitation Training and Research Hospital, Health Sciences University, 34303 Istanbul, Türkiye; berrak8@hotmail.com; 5 Department of Internal Medicine, Division of Geriatrics, Tekirdağ Dr. İsmail Fehmi Cumalıoğlu City Hospital, 59030 Tekirdağ, Türkiye; driremkirac@gmail.com; 6 Department of Internal Medicine, Faculty of Medicine, Istanbul Medipol University, 34810 Istanbul, Türkiye; esracokicli@hotmail.com; 7 Department of Medical Biochemistry, Faculty of Medicine, Istanbul Atlas University, 34403 Istanbul, Türkiye; nailemisirlioglu@gmail.com (N.F.M.); huzun59@hotmail.com (H.U.); 8 Department of Physiology, Faculty of Medicine, Istanbul Atlas University, 34403 Istanbul, Türkiye; gdincsimsek@yahoo.com

**Keywords:** Hashimoto’s thyroiditis, MOTS-c, mitochondria-derived peptides, autoimmune thyroid disease, immuno-metabolism, mitochondrial dysfunction

## Abstract

**Background**: Hashimoto’s thyroiditis (HT) is a common autoimmune disorder characterized by chronic inflammation and metabolic alterations. Mitochondria-derived peptides (MDPs), particularly mitochondrial open-reading frame of the 12S rRNA-c (MOTS-c), have emerged as key regulators of cellular metabolism, insulin sensitivity, oxidative stress, and inflammatory responses. This study aimed to investigate the association between circulating MOTS-c levels and HT and to explore its potential role in thyroid autoimmunity and metabolic regulation. **Methods**: In this cross-sectional study, patients diagnosed with HT (*n*: 90) were compared with age- and sex-matched healthy controls (*n*: 90). **Results**: A total of 180 participants were included, comprising 90 patients with HT and 90 age- and sex-matched healthy controls. Circulating MOTS-c levels were significantly lower in patients with HT compared to controls (*p* < 0.001). MOTS-c levels demonstrated significant inverse correlations with body mass index, fasting glucose, HbA1c, HOMA-IR, thyroid-stimulating hormone, C-reactive protein, and thyroid autoantibody levels (all *p* < 0.05). In subgroup analyses, these associations remained significant within the HT cohort, particularly for HOMA-IR and thyroid autoantibodies. Multivariable regression analysis identified HT (β = −30.04, *p* < 0.001) and HOMA-IR (β = −0.85, *p* < 0.001) as independent determinants of reduced circulating MOTS-c levels. Levothyroxine (LT4) use was not associated with significant differences in MOTS-c concentrations. **Conclusions**: Circulating MOTS-c levels are markedly reduced in patients with HT and are independently associated with insulin resistance and autoimmune burden. These findings suggest that impaired mitochondrial signaling may play a role in the pathophysiology of thyroid autoimmunity and highlight MOTS-c as a promising biomarker linking metabolic dysfunction and immune dysregulation.

## 1. Introduction

Hashimoto’s thyroiditis (HT) is a chronic autoimmune thyroid disorder characterized by lymphocytic infiltration and the presence of thyroid-specific autoantibodies, particularly anti-thyroid peroxidase and anti-thyroglobulin antibodies. It is the leading cause of hypothyroidism in iodine-sufficient regions and occurs predominantly in women. Beyond thyroid dysfunction, HT is recognized as a systemic condition associated with low-grade inflammation, oxidative stress, and metabolic disturbances [1,2,3].

**Figure 1:**
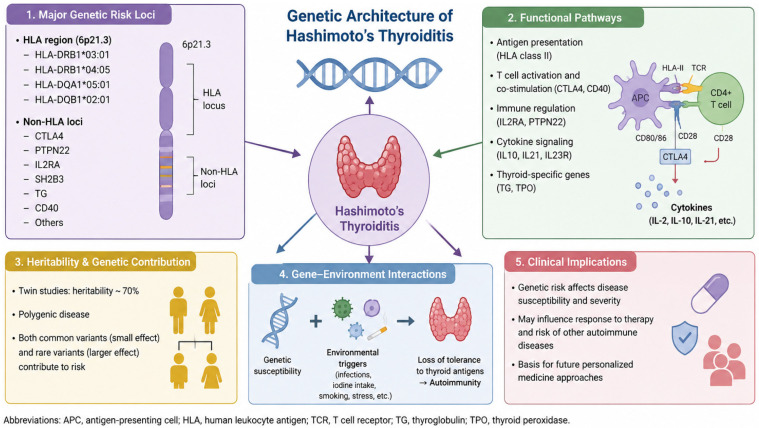
Genetic architecture and immunogenetic pathways involved in Hashimoto’s thyroiditis.

HT is a multifactorial autoimmune disorder involving complex interactions between genetic susceptibility, epigenetic regulation, and environmental triggers. In addition to its thyroid-specific effects, HT is associated with an increased predisposition to other autoimmune diseases. Figure 1 summarizes the major genetic susceptibility loci, immune regulatory pathways, and gene–environment interactions implicated in HT pathogenesis, including HLA-related and non-HLA immune-related genes [3,4,5,6,7].

Mitochondria, traditionally recognized for their role in energy production, are now considered key regulators of redox balance, stress responses, and immunometabolic signaling. This concept has been reinforced by the discovery of mitochondria-derived peptides encoded by short open-reading frames within mitochondrial DNA [8,9]. Among these peptides, mitochondrial open-reading frame of the 12S rRNA type-c (MOTS-c), identified in 2015, has emerged as an important regulator of metabolic homeostasis. MOTS-c is a 16-amino-acid peptide involved in glucose metabolism and insulin sensitivity through mechanisms including adenosine monophosphate–activated protein kinase (AMPK) activation and regulation of folate–purine metabolism. Under metabolic stress, MOTS-c may translocate to the nucleus and regulate adaptive gene expression, highlighting its role in mitonuclear communication [8,9,10,11]. In addition to its metabolic effects, MOTS-c exhibits anti-inflammatory and cytoprotective properties and has been implicated in oxidative stress regulation and immune modulation [11].

Altered circulating MOTS-c levels have been reported in several disorders, including obesity, insulin resistance (IR), type 1 diabetes mellitus (T1DM), chronic obstructive pulmonary disease (COPD), and multiple sclerosis (MS), suggesting a broader role in metabolic and inflammatory diseases [12,13,14,15]. These mechanisms are particularly relevant in HT, where oxidative stress, immune dysregulation, and metabolic alterations coexist [16]. Despite this biological relevance, the role of MOTS-c in autoimmune thyroid disease remains poorly understood, and no well-defined clinical studies have specifically evaluated circulating MOTS-c levels in patients with HT [11].

Therefore, this study aimed to evaluate circulating MOTS-c levels in patients with HT and to examine their associations with thyroid autoantibodies, thyroid function, and metabolic parameters. We hypothesized that MOTS-c levels would be altered in HT and reflect both autoimmune and metabolic burdens [10,11,17,18].

## 2. Materials and Methods

### 2.1. Ethical Approval

The study protocol was approved by the Health Sciences University Istanbul Kanuni Sultan Suleyman Training and Research Hospital Ethics Committee (number and date of approval: 125/28 May 2025). Written informed consent was obtained from both the patient and control groups following comprehensive verbal explanations.

### 2.2. Study Design and Population

This case–control study included a total of 180 participants, comprising 90 patients diagnosed with HT and 90 age- and sex-matched healthy controls. Patients were recruited from the outpatient endocrinology clinic. The diagnosis of HT was established according to currently accepted criteria, defined by the presence of thyroid-specific autoantibodies (anti-thyroid peroxidase and/or anti-thyroglobulin antibodies) and/or decreased echogenicity of the thyroid parenchyma on ultrasonography, in the appropriate clinical context [19]. Control subjects were selected from individuals without a history of thyroid disease, autoimmune disorders, or chronic systemic illness. Exclusion criteria for all participants included pregnancy, active infection, known malignancy, chronic inflammatory disease, liver or renal failure, and the use of medications that could influence metabolic or inflammatory parameters.

### 2.3. Clinical and Laboratory Assessments

Demographic and clinical data, including age, sex, height, weight, and levothyroxine (LT4) use, were obtained from medical records. Body mass index (BMI) was calculated as weight (kg) divided by height squared (m^2^) [20].

Fasting venous blood samples were obtained after an overnight fast of at least 8 h and collected into serum separator tubes under standardized pre-analytical conditions. Hemolyzed or visibly lipemic samples were excluded from the analysis. Serum aliquots were stored under controlled conditions at −80 °C, and repeated freeze–thaw cycles were avoided. All samples underwent only a single thawing procedure and were analyzed within the same assay batch to minimize inter-assay variability.

Routine biochemical parameters, including fasting glucose, lipid profile, liver enzymes, and creatinine, were measured using standard enzymatic methods on an automated analyzer (Cobas 8000, Roche Diagnostics, Mannheim, Germany). C-reactive protein (CRP), identified via immunoturbidimetric assay, was analyzed using the Roche Cobas 8000 c 702 analyzer (Roche Diagnostics, Mannheim, Germany). Glycated hemoglobin (HbA1c) levels were determined using high-performance liquid chromatography (HPLC) with an ARKRAY/ADAMS HA-8180V (ARKRAY Inc., Kyoto, Japan).

All samples were analyzed in duplicate within the same assay run. Thyroid function parameters, including thyroid-stimulating hormone (TSH), free triiodothyronine (fT3), free thyroxine (fT4), anti-thyroid peroxidase (anti-TPO), and anti-thyroglobulin (anti-TG) antibodies, were measured using an electrochemiluminescence immunoassay system (Elecsys, Roche Diagnostics, Mannheim, Germany) with commercially available DiaSorin assay kits (DiaSorin S.p.A., Saluggia, Italy) according to the manufacturers’ instructions.

Insulin was analyzed by electrochemiluminescence on a Roche Cobas E801 analyzer (Roche Diagnostics, Mannheim, Germany). Insulin resistance (IR) was estimated using the homeostasis model assessment of insulin resistance (HOMA-IR) index, calculated as fasting insulin (µIU/mL) × fasting glucose (mg/dL)/405 [21].

Internal quality assurance was performed using Lyphochek Immunoassay Plus Control Trilevel 370 (Bio-Rad Laboratories Inc., San Diego, CA, USA), while external quality assessment was maintained through participation in the EQAS Immunoassay Monthly Program BC75 (Bio-Rad Laboratories Inc., San Diego, CA, USA).

Serum MOTS-c levels were measured using a commercially available enzyme-linked immunosorbent assay (ELISA) kit (MyBioSource, Cat. No: MBS2088114, MyBioSource, Inc., San Diego, CA, USA) according to the manufacturer’s instructions. The analytical sensitivity (lower limit of detection) was defined by the manufacturer as the lowest concentration distinguishable from zero, calculated based on the mean optical density of 20 zero-standard replicates minus two standard deviations. The assay demonstrated high sensitivity and specificity for MOTS-c detection, with no significant cross-reactivity or interference from structurally related analogues. Each serum sample was measured in duplicate within the same assay run, and the mean value was used for statistical analysis. The assay performance characteristics included an intra-assay coefficient of variation (CV) of <10%, reflecting repeatability within the same assay run, and an inter-assay CV of <12%, reflecting reproducibility between different assay runs. The assay sensitivity was 0.93 ng/mL, with a measurable range of 2.47–200 ng/mL.

### 2.4. Statistical Analysis

All statistical analyses were performed using IBM SPSS Statistics for Windows, version 28.0 (IBM Corp., Armonk, NY, USA). The distribution of continuous variables was assessed using visual (histograms and Q–Q plots) and analytical methods (Shapiro–Wilk test). Continuous variables are presented as mean ± standard deviation or median (interquartile range), depending on data distribution, while categorical variables are expressed as counts and percentages. Between-group comparisons (HT vs. control) were performed using the independent samples *t*-test for normally distributed variables and the Mann–Whitney U test for non-normally distributed variables. Correlation analyses were performed to evaluate the relationships between circulating MOTS-c levels and clinical, metabolic, and immunological parameters. Pearson correlation analysis was used for normally distributed variables, whereas Spearman rank correlation analysis was applied for non-normally distributed variables. A two-tailed *p* value < 0.05 was considered statistically significant. False discovery rate (FDR) correction using the Benjamini–Hochberg procedure was also applied for multiple comparisons where appropriate. Receiver operating characteristic (ROC) curve analysis was performed to evaluate the discriminatory performance of circulating MOTS-C levels for identifying Hashimoto’s thyroiditis. BMI-adjusted analyses were also performed using multivariable linear regression models.

## 3. Results

### 3.1. Baseline Characteristics

A total of 180 participants were included, comprising 90 patients with HT and 90 age- and sex-comparable controls. There were no significant differences between groups in terms of age, sex distribution, or most anthropometric parameters (all *p* > 0.05). However, BMI was modestly higher in the Hashimoto group (*p* = 0.040) (Table 1).

Regarding metabolic parameters, fasting glucose and HbA1c levels were comparable between groups, whereas HOMA-IR was significantly lower in patients with HT (*p* = 0.004). Lipid profile and liver enzyme levels did not differ significantly.

As expected, thyroid-related parameters demonstrated marked differences. Patients with HT exhibited significantly higher TSH, anti-thyroid peroxidase, and anti-thyroglobulin antibody levels (all *p* < 0.001), while free T3 and free T4 levels were comparable.

**Figure 2:**
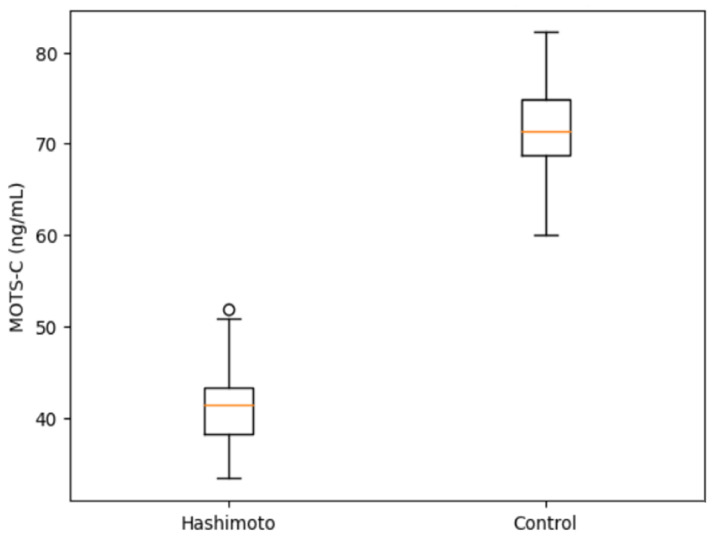
Distribution of MOTS-C levels in patients with Hashimoto’s thyroiditis and control subjects.

Importantly, circulating MOTS-C levels were profoundly reduced in the Hashimoto group compared to controls (median: 41.33 vs. 71.30 ng/mL, *p* < 0.001), indicating a substantial alteration in mitochondrial-derived peptide signaling (Table 1, Figure 2).

Additionally, BMI-adjusted analyses confirmed that circulating MOTS-c levels remained significantly lower in patients with HT compared with controls (adjusted *p* < 0.001, Supplementary Table S1).

### 3.2. Correlation Analyses

In the overall cohort, circulating MOTS-C levels demonstrated significant inverse correlations with key metabolic and inflammatory parameters, including BMI (r = −0.32), fasting glucose (r = −0.18), HbA1c (r = −0.21), and HOMA-IR (r = −0.48) (all *p* ≤ 0.015). Additionally, strong negative associations were observed with thyroid-related markers, particularly TSH (r = −0.41), anti-thyroid peroxidase (r = −0.52), and anti-thyroglobulin antibodies (r = −0.47) (all *p* < 0.001). A modest inverse correlation was also noted with CRP (r = −0.26, *p* = 0.001). In the control subgroup, most correlations between MOTS-C levels and metabolic or thyroid-related parameters were not statistically significant, except for weak positive correlations with BMI and free T4 (Table 2).

Subgroup analysis restricted to patients with HT revealed even stronger associations. MOTS-C levels were inversely correlated with HOMA-IR (r = −0.55), TSH (r = −0.46), anti-thyroid peroxidase (r = −0.58), and anti-thyroglobulin antibodies (r = −0.50) (all *p* < 0.001). These findings highlight a robust link between reduced MOTS-C levels and both metabolic dysregulation and autoimmune burden.

After false discovery rate correction using the Benjamini–Hochberg procedure, the principal associations remained statistically significant (Supplementary Table S2).

**Figure 3:**
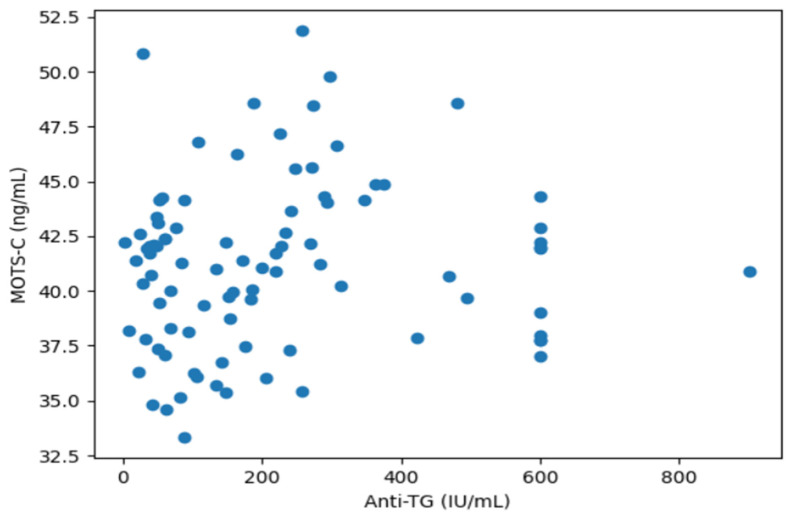
Relationship between anti-thyroglobulin antibody (Anti-TG) levels and circulating MOTS-C concentrations in patients with Hashimoto’s thyroiditis.

Furthermore, scatter plot analysis revealed a significant inverse relationship between anti-thyroglobulin antibody levels and MOTS-C concentrations (ρ = −0.50, *p* < 0.001), reinforcing the association between mitochondrial signaling and autoimmune activity (Figure 3).

### 3.3. Multivariable Regression Analysis

In unadjusted analysis, the presence of HT was strongly associated with lower MOTS-C levels (β = −30.26, *p* < 0.001). This association remained highly significant after sequential adjustment for demographic and metabolic confounders (Table 3).

In the fully adjusted model, HT remained an independent determinant of reduced MOTS-C levels (β = −30.04, 95% CI: −31.43 to −28.65, *p* < 0.001). Among covariates, only HOMA-IR independently predicted MOTS-C concentrations (β = −0.85, *p* < 0.001), whereas age, sex, body mass index, TSH, CRP, and LT4 use were not significant contributors.

Notably, collinearity diagnostics confirmed the robustness of the model, with all variance inflation factor values below 2.

### 3.4. ROC Analysis

**Figure 4:**
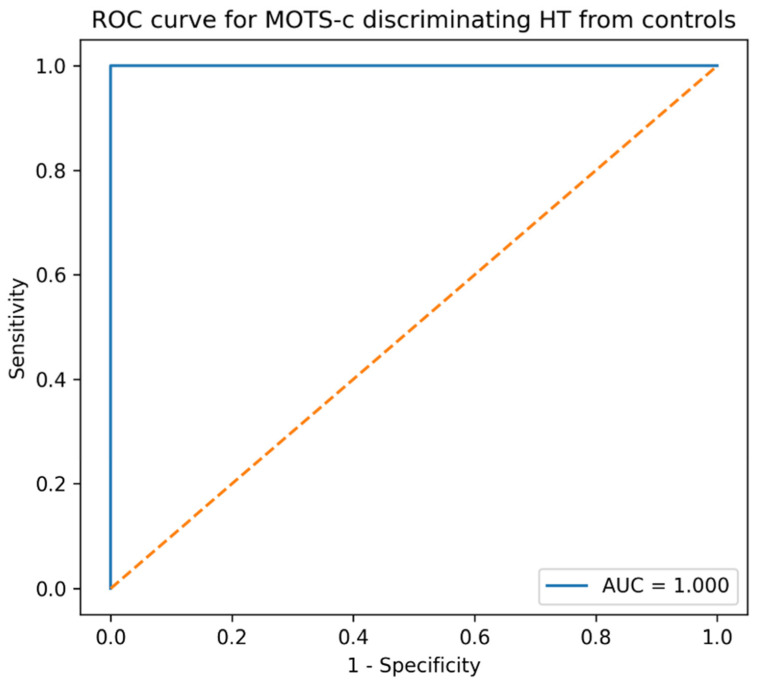
Receiver operating characteristic (ROC) curve analysis of circulating MOTS-C levels for discrimination of Hashimoto’s thyroiditis. The blue line represents the ROC curve for circulating MOTS-C levels, whereas the orange dashed diagonal line indicates the line of no discrimination (reference line).

Receiver operating characteristic (ROC) curve analysis demonstrated excellent discriminatory performance of circulating MOTS-c levels for identifying Hashimoto’s thyroiditis, with an area under the curve (AUC) of 1.00. The optimal cutoff value determined using the Youden index was 51.88 ng/mL, yielding 100% sensitivity and 100% specificity (Figure 4).

### 3.5. Impact of Levothyroxine (LT4) Treatment

Within the Hashimoto subgroup, no significant differences were observed between LT4 users and non-users in terms of MOTS-C levels (*p* = 0.74) or other metabolic parameters. This suggests that thyroid hormone replacement therapy was not associated with measurable alterations in circulating MOTS-C concentrations in this cross-sectional analysis.

## 4. Discussion

In this study, we demonstrated that circulating MOTS-c levels are markedly reduced in patients with HT compared with healthy controls. This is the principal and most robust finding of our analysis. This difference remained highly significant after comprehensive adjustment for demographic, metabolic, and inflammatory confounders, indicating that the association between HT and decreased MOTS-c is independent and not merely a reflection of metabolic status. Moreover, MOTS-c levels exhibited consistent and significant inverse correlations with markers of thyroid autoimmunity (anti-TPO and anti-TG), thyroid function (TSH), and metabolic parameters, particularly insulin resistance (HOMA-IR), suggesting a potential integrative role at the intersection of immune dysregulation and metabolic homeostasis. Collectively, these findings support the concept that impaired mitochondrial-derived peptide signaling may represent a novel pathophysiological component of HT, linking mitochondrial dysfunction to both autoimmune activity and metabolic alterations. Although ROC analysis demonstrated excellent discriminatory performance in the present cohort, this finding should be interpreted cautiously and requires external validation in larger independent populations.

Histopathologically, HT is characterized by diffuse lymphocytic infiltration, formation of lymphoid follicles with germinal centers, follicular destruction, and varying degrees of fibrosis and Hürthle cell metaplasia. In addition to the classical form, several histopathological variants, including fibrous and IgG4-related forms, have been described and may differ in inflammatory activity and clinical progression. Despite the availability of established histopathological criteria, interpretation may vary among clinicians and pathologists, highlighting the heterogeneous nature of HT [22,23].

In present study, when the baseline characteristics of the study population are considered, the two groups were largely comparable in terms of demographic and most metabolic parameters, supporting the internal validity of the comparisons. Age, sex distribution, anthropometric measures (except BMI), glycemic indices, lipid profile, liver enzymes, renal function, and CRP levels did not differ significantly between patients with HT and controls. Notably, BMI was modest but significantly higher in the Hashimoto group, which is consistent with previous reports suggesting a tendency toward increased adiposity in autoimmune thyroid disease [24,25,26,27,28,29]. Most studies suggest a positive association between serum TSH levels and BMI, indicating a link between thyroid function and adiposity. Large-scale analyses and meta-analyses have shown that higher TSH levels, even within the normal range, are associated with increased BMI and a higher risk of overt or subclinical hypothyroidism, particularly in individuals with HT. However, this relationship is not entirely consistent, as some large cross-sectional studies have reported no significant association between TSH and BMI [24,25,26,27,28,29]. In contrast, HOMA-IR levels were significantly lower in patients with HT, despite similar fasting glucose and HbA1c levels. This finding contrasts with most previous reports suggesting increased insulin resistance in HT [30,31,32]. Insulin resistance, defined as a reduced responsiveness of peripheral tissues such as muscle, liver, and adipose tissue to insulin, is a key disturbance in glucose homeostasis and contributes to the development of type 2 diabetes mellitus through compensatory hyperinsulinemia [30]. Thyroid function is known to influence insulin sensitivity not only in patients with diabetes but also in individuals with normal glucose tolerance [31,32]. Hypothyroidism has been associated with impaired glucose metabolism, while restoration of an euthyroid state may improve insulin sensitivity [33]. The discrepancy observed in our study may be explained by the predominantly euthyroid or subclinical disease status of our cohort, as well as the relatively high proportion of patients receiving LT4 therapy, both of which may enhance insulin sensitivity. Although BMI was modestly higher in the HT group, fasting insulin levels were not proportionally increased, which may partly explain the unexpectedly lower HOMA-IR values observed in this cohort. Moreover, the relationship between thyroid autoimmunity and insulin resistance is likely complex and not strictly linear, potentially varying according to disease stage, treatment status, and individual metabolic characteristics. These findings suggest that insulin resistance in HT may not be uniformly elevated and should be interpreted within the broader clinical context.

As anticipated, thyroid-related parameters demonstrated marked differences, with significantly higher TSH, anti-TPO, and anti-TG levels in patients with HT, while free T3 and free T4 levels remained comparable, reflecting a predominantly euthyroid or subclinical disease state [34]. Importantly, the absence of significant differences in systemic inflammatory markers such as CRP suggests that the observed alterations are not driven by overt systemic inflammation. Collectively, these findings indicate that, aside from thyroid-specific autoimmunity and a modest increase in BMI, the study groups were well balanced, thereby strengthening the interpretation that the observed differences in mitochondrial-derived peptide levels are likely related to disease-specific mechanisms rather than confounding metabolic or inflammatory factors. The study by Bozdag and Gundogan Bozdag [35] demonstrated that HT is associated with low-grade systemic inflammation, even when conventional markers such as CRP remain within normal limits. This finding is consistent with our results and suggests that subtle inflammatory changes in HT may require more sensitive biomarkers for accurate detection.

Our current findings suggest that the low MOTS-c levels detected in HT may be biologically significant, as MOTS-c has been shown in experimental and clinical studies to be closely associated with the regulation of energy homeostasis, insulin sensitivity, oxidative stress response, and inflammation [36,37,38,39]. However, evidence regarding circulating MOTS-c levels in human obesity and related metabolic conditions is still scarce and inconsistent. Early studies have suggested that obese adolescents exhibit lower MOTS-c levels compared to lean individuals, with reported inverse associations between MOTS-c concentrations and indicators of adiposity and insulin resistance, including BMI and HOMA-IR [13,15]. In the first descriptive study, MOTS-c was reported to reduce obesity and insulin resistance in high-fat diet conditions, and human studies have shown that circulating MOTS-c levels are associated with metabolic status and insulin sensitivity [40,41]. On the other hand, it is well known that chronic inflammation and oxidative stress play a central role in the pathogenesis of HT [42]. Therefore, the inverse relationship of MOTS-c with anti-TPO, anti-TG, TSH, and HOMA-IR in our study suggests that the decrease in MOTS-c may reflect not only a metabolic epiphenomenon but also a mitochondrial signaling disorder associated with autoimmune burden and impaired cellular stress response. To the best of current knowledge and based on the available peer-reviewed literature, no previous original clinical study has directly assessed circulating MOTS-c levels in HT; therefore, the present findings may represent a novel and original contribution to the literature. The observed reduction in circulating MOTS-c levels in patients with HT may be biologically plausible considering the established metabolic and immunomodulatory functions of this mitochondrial-derived peptide. Experimental evidence has demonstrated that MOTS-c plays a critical role in maintaining metabolic homeostasis by enhancing insulin sensitivity, regulating glucose utilization, and attenuating oxidative stress pathways [38]. In human studies, circulating MOTS-c concentrations have been shown to correlate with insulin sensitivity, particularly in metabolically healthy individuals [40]. Given that HT is characterized by chronic low-grade inflammation and increased oxidative stress, both of which contribute to tissue damage and autoimmune activation, the inverse associations observed in our study between MOTS-c and thyroid autoantibodies, TSH, and HOMA-IR suggest that reduced MOTS-c may reflect impaired mitochondrial adaptive responses in this setting. Indeed, oxidative stress has been consistently implicated as a central mechanism in the pathogenesis of autoimmune thyroid disorders [43], and metabolic disturbances such as obesity-related oxidative imbalance may further exacerbate this process [44]. In this context, the consistent negative correlations between MOTS-c and both autoimmune and metabolic parameters observed in our cohort support the hypothesis that mitochondrial signaling pathways may represent a mechanistic link between immune dysregulation and metabolic alterations in HT. The role of MOTS-c in regulating autoimmune tolerance may extend beyond type 1 diabetes, as regulatory T cells are critically involved in a broad spectrum of autoimmune diseases, including HT, multiple sclerosis, rheumatoid arthritis, and Graves’ disease. This broader implication warrants further investigation [11].

LT4 use was significantly more common in the HT group, reflecting standard clinical management. However, subgroup analyses demonstrated no significant differences in circulating MOTS-c levels or metabolic parameters between LT4 users and non-users within the HT cohort. These findings suggest that LT4 therapy was not associated with measurable alterations in circulating MOTS-c levels in this cross-sectional analysis. Nevertheless, these observations should be interpreted cautiously, as the study design does not allow conclusions regarding treatment efficacy, mitochondrial function, or causal metabolic effects [45]. Further longitudinal and interventional studies are needed to clarify the potential relationship between thyroid hormone replacement and mitochondrial-derived peptide signaling.

The thyroid gland contains the highest selenium concentration per gram of tissue among human organs, reflecting the critical role of selenoproteins in thyroid physiology and antioxidant defense. Selenoproteins, including glutathione peroxidases, thioredoxin reductases, and iodothyronine deiodinases, contribute to thyroid hormone metabolism and protect thyrocytes against oxidative stress generated during hormone synthesis. Increasing amounts of evidence also suggests that impaired selenium status and altered selenoprotein activity may contribute to thyroid autoimmunity and the pathogenesis of HT through dysregulated redox balance and immune responses [46]. Recent evidence also suggests that impaired antioxidant pathways may contribute to mitochondrial dysfunction and altered mitochondrial-derived peptide signaling, including MOTS-c, in autoimmune and metabolic disorders. Therefore, reduced MOTS-c levels observed in HT may partly reflect disrupted redox homeostasis and impaired cellular protective mechanisms associated with thyroid autoimmunity [36].

Thyroid peroxidase is a heme-dependent enzyme, and iron deficiency may impair thyroid hormone synthesis by reducing thyroid peroxidase activity. Recent evidence has also demonstrated a significant association between iron deficiency and altered thyroid function, particularly in autoimmune thyroid disorders such as HT. Therefore, impaired iron status may contribute to oxidative stress, thyroid dysfunction, and autoimmune activity in HT [47].

Recent evidence suggests that the gut microbiome plays an important role in thyroid hormone metabolism and immune regulation. Gut dysbiosis may impair intestinal barrier integrity, alter micronutrient absorption, and promote chronic inflammation, thereby contributing to autoimmune thyroid diseases such as HT. In this context, alterations in microbiota-related metabolic and immune pathways may also be associated with mitochondrial dysfunction and impaired cellular stress responses [48].

### 4.1. Strengths and Limitations

This study has several important strengths. Firstly, it is, to the best of our knowledge, the first clinical investigation to evaluate circulating MOTS-c levels in patients with HT, providing novel insight into the potential role of mitochondrial-derived peptides in thyroid autoimmunity. Secondly, the relatively well-matched control group and the comprehensive assessment of metabolic, inflammatory, and thyroid-related parameters strengthen the internal validity of the findings. Thirdly, the use of multivariable regression analyses allows for robust adjustment of potential confounders, supporting the independence of the observed associations.

However, several limitations should be acknowledged. First, the cross-sectional design precludes causal inference; therefore, it cannot be determined whether reduced MOTS-c levels are a cause or a consequence of HT. Second, the single-center nature of the study may limit the generalizability of the findings. In addition, the lack of longitudinal follow-up prevented the evaluation of temporal changes in MOTS-c levels during disease progression or treatment. Detailed longitudinal data regarding disease duration and quantitative assessment of thyroid tissue destruction were also not consistently available; therefore, these parameters could not be comprehensively evaluated in the present study. Although major confounding factors were considered, unmeasured variables such as dietary habits, physical activity, and detailed body composition parameters could not be fully assessed. Furthermore, protease inhibitors were not used during sample processing, which may have affected peptide stability and represents a potential methodological limitation. Finally, the use of a single commercially available ELISA platform without external validation may have influenced the absolute circulating MOTS-c concentrations and may limit broader comparability of the findings.

### 4.2. Clinical Implications

The present findings suggest that MOTS-c may represent a potential biomarker reflecting the interaction between metabolic regulation and autoimmune activity in HT. Although no significant association was observed between LT4 therapy and circulating MOTS-c levels, this finding should be interpreted cautiously given the cross-sectional and non-randomized design of the study. Therefore, no conclusions can be drawn regarding the effects of LT4 therapy on mitochondrial function or metabolic regulation. Further prospective and interventional studies are required to clarify the clinical significance of MOTS-c and its potential relationship with thyroid hormone replacement therapy.

### 4.3. Future Directions

Future studies should focus on longitudinal and mechanistic investigations to better clarify the relationship between reduced MOTS-c levels and the development or progression of HT. Prospective studies may help determine whether circulating MOTS-c levels are associated with disease activity, metabolic alterations, or therapeutic responses over time. In addition, experimental research exploring the potential role of MOTS-c in oxidative stress regulation, immune modulation, and mitochondrial signaling may provide further insight into its biological relevance in thyroid autoimmunity. Finally, integrating advanced metabolic phenotyping and multi-omics approaches may contribute to a more comprehensive understanding of the interplay between mitochondrial function, metabolism, and autoimmune thyroid disease.

## 5. Conclusions

Circulating MOTS-c levels were significantly reduced in patients with HT and were independently associated with markers of thyroid autoimmunity and metabolic regulation. These findings support a potential relationship between mitochondrial-derived peptide signaling, immune dysregulation, and metabolic alterations in HT. However, given the cross-sectional design of the study, causal inferences cannot be established. Further longitudinal and mechanistic studies are needed to clarify the biological and potential clinical significance of MOTS-c in autoimmune thyroid disease.

## Figures and Tables

**Table 1:** Demographic, clinical, and laboratory characteristics of patients with Hashimoto’s thyroiditis and controls.

Variable	Hashimoto’s Thyroiditis (*n* = 90)	Control (*n* = 90)	*p* Value
**Age (years) †**	42.5 (32.0–48.0)	35.5 (27.0–46.0)	0.071
Female sex, *n* (%) ‡	74 (82.2%)	70 (77.8%)	0.47
Height (cm) †	160.0 (155.0–165.0)	162.0 (156.0–167.0)	0.21
Weight (kg) †	68.0 (58.0–75.0)	64.0 (56.0–72.0)	0.18
BMI (kg/m^2^) †	26.99 (23.96–29.41)	24.59 (22.16–28.75)	**0.040**
HbA1c (%) †	5.3 (5.1–5.6)	5.2 (5.0–5.5)	0.22
Fasting glucose (mg/dL) †	92.0 (87.0–95.75)	89.5 (84.0–94.75)	0.175
Insulin (µIU/mL) †	5.87 (3.37–12.24)	8.42 (6.80–10.00)	**0.004**
HOMA-IR †	1.45 (0.83–3.02)	2.08 (1.68–2.47)	**0.004**
Total cholesterol (mg/dL) †	201.0 (179.0–223.0)	196.0 (172.0–218.0)	0.31
LDL cholesterol (mg/dL) †	124.0 (103.0–147.0)	118.0 (99.0–141.0)	0.28
Triglycerides (mg/dL) †	118.0 (89.0–151.0)	111.0 (86.0–145.0)	0.33
Uric acid (mg/dL) †	4.6 (3.9–5.3)	4.4 (3.8–5.1)	0.29
AST (U/L) †	18.0 (15.0–22.0)	17.0 (14.0–21.0)	0.41
ALT (U/L) †	17.0 (13.0–23.0)	16.0 (12.0–22.0)	0.48
GGT (U/L) †	15.0 (11.0–21.0)	14.0 (10.0–20.0)	0.52
Creatinine (mg/dL) †	0.71 (0.64–0.81)	0.73 (0.66–0.82)	0.44
TSH (mIU/L) †	2.76 (1.90–3.85)	1.64 (1.05–2.20)	**<0.001**
Free T3 (pg/mL) †	2.98 (2.40–3.42)	3.05 (2.51–3.51)	0.19
Free T4 (ng/dL) †	1.26 (1.09–1.42)	1.29 (1.12–1.45)	0.27
Anti-TPO (IU/mL) †	186.0 (72.0–412.0)	9.2 (6.4–13.8)	**<0.001**
Anti-TG (IU/mL) †	132.0 (58.0–268.0)	12.5 (7.1–18.3)	**<0.001**
CRP (mg/L) †	1.42 (0.78–2.31)	1.18 (0.62–2.04)	0.17
Levothyroxine (LT4) use, *n* (%) ‡	56 (62.2%)	0 (0%)	**<0.001**
MOTS-C (ng/mL) †	41.33 (38.15–43.28)	71.30 (68.69–74.80)	**<0.001**

† Compared using the Mann–Whitney U test. ‡ Compared using the χ^2^ test or Fisher’s exact test, as appropriate. Continuous variables are presented as median (interquartile range). Categorical variables are presented as number (%). Bold values indicate statistically significant results (*p* < 0.05). **Abbreviations: BMI**—body mass index; **HOMA-IR**—homeostasis model assessment of insulin resistance; **TSH**—thyroid-stimulating hormone; **Anti-TPO**—anti-thyroid peroxidase antibody; **Anti-TG**—anti-thyroglobulin antibody; **CRP**—C-reactive protein; **HbA1c**—glycated hemoglobin; **LDL**—low-density lipoprotein cholesterol; **AST**—aspartate aminotransferase; **ALT**—alanine aminotransferase; **GGT**—gamma-glutamyl transferase; **MOTS-C**—mitochondrial open-reading frame of the 12S rRNA type-c peptide.

**Table 2:** Correlation of circulating MOTS-c levels with metabolic and thyroid-related parameters.

**(A) Whole Cohort (*n* = 180).**			
**Variable**	**Spearman’s ρ**	**95% CI**	***p* Value**
Age (years)	−0.09	−0.23 to 0.06	0.21
BMI (kg/m^2^)	−0.32	−0.45 to −0.18	**<0.001**
Fasting glucose (mg/dL)	−0.18	−0.32 to −0.03	**0.015**
HbA1c (%)	−0.21	−0.35 to −0.06	**0.006**
HOMA-IR	−0.48	−0.59 to −0.35	**<0.001**
TSH (mIU/L)	−0.41	−0.53 to −0.27	**<0.001**
Free T3 (pg/mL)	0.12	−0.03 to 0.26	0.10
Free T4 (ng/dL)	0.09	−0.06 to 0.24	0.19
Anti-TPO (IU/mL)	−0.52	−0.62 to −0.40	**<0.001**
Anti-TG (IU/mL)	−0.47	−0.58 to −0.34	**<0.001**
CRP (mg/L)	−0.26	−0.39 to −0.12	**0.001**
**(B) Hashimoto’s Thyroiditis Subgroup (*n* = 90).**			
**Variable**	**Spearman’s ρ**	**95% CI**	***p* Value**
Age (years)	−0.11	−0.31 to 0.10	0.29
BMI (kg/m^2^)	−0.28	−0.46 to −0.08	**0.008**
Fasting glucose (mg/dL)	−0.22	−0.42 to −0.01	**0.036**
HbA1c (%)	−0.24	−0.44 to −0.03	**0.024**
HOMA-IR	−0.55	−0.70 to −0.36	**<0.001**
TSH (mIU/L)	−0.46	−0.63 to −0.27	**<0.001**
Anti-TPO (IU/mL)	−0.58	−0.72 to −0.40	**<0.001**
Anti-TG (IU/mL)	−0.50	−0.66 to −0.31	**<0.001**
CRP (mg/L)	−0.31	−0.50 to −0.10	**0.004**
**(C) Control Subgroup (*n* = 90).**			
**Variable**	**Spearman’s ρ**	**95% CI**	***p* Value**
Age (years)	−0.03	−0.24 to 0.18	0.756
BMI (kg/m^2^)	0.23	0.02 to 0.41	**0.033**
Fasting glucose (mg/dL)	0.08	−0.13 to 0.29	0.432
HbA1c (%)	0.03	−0.18 to 0.24	0.777
HOMA-IR	0.10	−0.11 to 0.30	0.354
TSH (mIU/L)	0.05	−0.16 to 0.25	0.640
Free T3 (pg/mL)	0.18	−0.03 to 0.38	0.085
Free T4 (ng/dL)	0.22	0.02 to 0.41	**0.033**
Anti-TPO (IU/mL)	0.14	−0.07 to 0.34	0.177
Anti-TG (IU/mL)	0.00	−0.21 to 0.21	0.987
CRP (mg/L)	0.06	−0.15 to 0.26	0.598

Bold values indicate statistically significant results (*p* < 0.05). **Abbreviations: BMI**—body mass index; **HOMA-IR**—homeostasis model assessment of insulin resistance; **TSH**—thyroid-stimulating hormone; **CRP**—C-reactive protein; **MOTS-C**—mitochondrial open-reading frame of the 12S rRNA type-c peptide.

**Table 3:** Multivariable linear regression analysis for determinants of circulating MOTS-c levels. Dependent variable: MOTS-c (ng/mL).

**Model 1. Unadjusted.**			
**Variable**	**β (SE)**	**95% CI**	***p* Value**
Hashimoto’s thyroiditis	−30.26 (0.72)	−31.68 to −28.84	**<0.001**
**Model 2. Adjusted for Age and Sex.**			
**Variable**	**β (SE)**	**95% CI**	***p* Value**
Hashimoto’s thyroiditis	−30.21 (0.71)	−31.60 to −28.82	**<0.001**
Age (years)	−0.04 (0.03)	−0.10 to 0.02	0.19
Female sex	−0.58 (0.62)	−1.80 to 0.64	0.35
**Model 3. Metabolic Adjustment.**			
**Variable**	**β (SE)**	**95% CI**	***p* Value**
Hashimoto’s thyroiditis	−30.13 (0.70)	−31.50 to −28.76	**<0.001**
Age (years)	−0.03 (0.03)	−0.09 to 0.03	0.31
Sex (Female)	−0.51 (0.61)	−1.70 to 0.68	0.41
Body mass index (kg/m^2^)	−0.12 (0.07)	−0.26 to 0.01	0.097
HOMA-IR	−0.88 (0.19)	−1.25 to −0.51	**<0.001**
**Model 4. Fully Adjusted Model.**			
**Variable**	**β (SE)**	**95% CI**	***p* Value**
**Hashimoto’s thyroiditis**	−30.04 (0.71)	−31.43 to −28.65	**<0.001**
Age (years)	−0.03 (0.03)	−0.09 to 0.03	0.34
Female sex	−0.49 (0.61)	−1.69 to 0.71	0.43
Body mass index (kg/m^2^)	−0.10 (0.07)	−0.24 to 0.03	0.14
HOMA-IR	−0.85 (0.20)	−1.24 to −0.46	**<0.001**
TSH (mIU/L)	−0.18 (0.12)	−0.41 to 0.05	0.12
CRP (mg/L)	−0.21 (0.17)	−0.55 to 0.13	0.22
Levothyroxine (LT4) use	−0.67 (0.63)	−1.90 to 0.56	0.29

Bold values indicate statistically significant results (*p* < 0.05). Linear regression analyses were performed to identify independent determinants of MOTS-C levels. β coefficients represent unstandardized estimates. Model 1 was unadjusted; Model 2 was adjusted for age and sex; Model 3 also adjusted for BMI and HOMA-IR; Model 4 further adjusted for TSH, CRP, and levothyroxine use.

## Data Availability

The datasets used and analyzed in this study are available from the corresponding author upon reasonable request.

## Supplementary Materials

The following supporting information can be downloaded at: https://www.mdpi.com/article/10.3390/jcm15114002/s1, Table S1: BMI-adjusted analysis of circulating MOTS-c levels in patients with Hashimoto’s thyroiditis and controls; Table S2: False discovery rate (FDR)-adjusted *p* values for correlation analyses in the whole cohort.
