# Supplementary material for: Reduced Circulating MOTS-c Levels in Hashimoto’s Thyroiditis Reflect Integrated Autoimmune and Metabolic Dysregulation: A Cross-Sectional Study
Source: J Clin Med. 2026 May 22;15(11):4002. doi: 10.3390/jcm15114002 (PMC13257931; doi:10.3390/jcm15114002)
Supplement: Supplementary file 1 [file jcm-15-04002-s001.zip › jcm-4298842-supplementary.pdf]

Supplementary Table S1. BMI-adjusted analysis of circulating MOTS-c levels in patients with Hashimoto's thyroiditis and controls.

**Variable**	**Unadjusted p value**	**BMI-adjusted p value**
MOTS-C (ng/mL)	<0.001	<0.001

BMI-adjusted analysis was performed using multivariable linear regression analysis. After adjustment for BMI, circulating MOTS-c levels remained significantly lower in patients with Hashimoto's thyroiditis compared with controls. BMI, body mass index; MOTS-C, mitochondrial open reading frame of the 12S rRNA type-c peptide.

Supplementary Table S2. False discovery rate (FDR)-adjusted p values for correlation analyses in the whole cohort.

**Variable**	**Raw p value**	**FDR-adjusted p value**
Age	0.21	0.210
BMI	<0.001	0.001
Fasting glucose	0.015	0.021
HbA1c	0.006	0.009
HOMA-IR	<0.001	0.001
TSH	<0.001	0.001
Free T3	0.10	0.122
Free T4	0.19	0.209
Anti-TPO	<0.001	0.001
Anti-TG	<0.001	0.001
CRP	0.001	0.002

False discovery rate correction was performed using the Benjamini–Hochberg procedure. The principal associations between circulating MOTS-c levels and metabolic and autoimmune parameters remained statistically significant after correction.

Abbreviations: BMI, body mass index; HOMA-IR, homeostasis model assessment of insulin resistance; TSH, thyroid-stimulating hormone; CRP, C-reactive protein; MOTS-C, mitochondrial open reading frame of the 12S rRNA type-c peptide.
